# Nanocellulose Composite Films in Food Packaging Materials: A Review

**DOI:** 10.3390/polym16030423

**Published:** 2024-02-02

**Authors:** Yanting Xu, Zhenzeng Wu, Ao Li, Nairong Chen, Jiuping Rao, Qinzhi Zeng

**Affiliations:** 1 Postgraduate Department, Minjiang University, No. 200, Xiyuangong Road, Fuzhou 350108, China; ytxu92@163.com; 2 The College of Ecology and Resource Engineering, Wuyi University, No. 16, Wuyi Avenue, Wuyishan 354300, China; zhenzeng.wu@wuyiu.edu.cn; 3 College of Material Engineering, Fujian Agriculture and Forestry University, 15 Shangxiadian Road, Fuzhou 350002, China; aoli@fafu.edu.cn (A.L.); fafucnr@163.com (N.C.)

**Keywords:** nanocellulose, nanocellulose composite films, preparation, food packaging, application

## Abstract

Owing to the environmental pollution caused by petroleum-based packaging materials, there is an imminent need to develop novel food packaging materials. Nanocellulose, which is a one-dimensional structure, has excellent physical and chemical properties, such as renewability, degradability, sound mechanical properties, and good biocompatibility, indicating promising applications in modern industry, particularly in food packaging. This article introduces nanocellulose, followed by its extraction methods and the preparation of relevant composite films. Meanwhile, the performances of nanocellulose composite films in improving the mechanical, barrier (oxygen, water vapor, ultraviolet) and thermal properties of food packaging materials and the development of biodegradable or edible packaging materials in the food industry are elaborated. In addition, the excellent performances of nanocellulose composites for the packaging and preservation of various food categories are outlined. This study provides a theoretical framework for the development and utilization of nanocellulose composite films in the food packaging industry.

## 1. Introduction

Food packaging plays an indispensable role in the food industry chain, serving food storage and transport by preventing chemical contamination and enhancing shelf life to ensure food security [1,2]. Previously, petroleum-based packaging materials were widely applied in the food packaging industry because of their strong plasticity, good mechanical and physical functions, ease of production, and low cost. However, these materials are not prone to degradation or available for recycling after use, seriously polluting the environment [3]. With the continuous promotion of ecological civilization, the search for renewable and biodegradable natural materials to replace petroleum-based products has gained much attention in academic circles in recent years.

Cellulose is a linear biopolymer and the most abundant organic material on earth. It is widely presented in higher plants, and can be acquired from marine animals, agricultural residues, industrial waste bacteria, and algae [4,5,6,7]. Natural cellulose has been widely employed for the application of composite materials owing to its advantages, such as low cost, degradability, and remarkable compatibility after modification. Nanocellulose is prepared by a series of methods, including chemical and mechanical treatments, as well as enzymatic hydrolysis after extracting cellulose from raw materials, and its diameter does not exceed 100 nm. Compared with ordinary cellulose, nanocellulose presents a variety of features, including a high Young’s modulus, high crystallinity, high specific surface area, high tensile strength, and other special traits, suggesting promising prospects for application in the fields of papermaking, biomedicine, materials packaging, etc.

Recently, experts worldwide have conducted a host of studies on the preparation, structure, performance characteristics, and applications of nanocellulose and nanocellulose-based composites [8,9,10,11,12]. With regard to composite films used in food packaging, nanocellulose-based composites are renowned for improving the mechanical and barrier properties of biopolymers. Additionally, they can be used as carriers of active substances such as antioxidants and antibacterial agents to extend the shelf life of food products [13,14], and thus have promising prospects.

To promote the understanding of the role of nanocellulose and nanocellulose composites in food packaging films and provide a reference for the development of novel nanocellulose composite films for food packaging, the resources, characteristics, classification, and preparation of nanocellulose are described in detail in this paper. Simultaneously, the preparation, properties, and applications of nanocellulose composite films in food packaging are reviewed. Finally, the safety issues and future prospects of nanocellulose-based food packaging are also discussed.

## 2. Classification and Preparation of Nanocellulose

### 2.1. Cellulose and Nanocellulose

**Figure 1:**
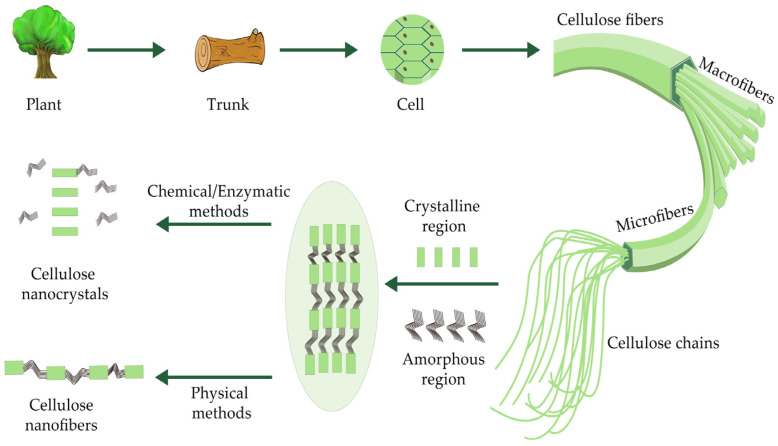
Schematic illustration of nanocellulose preparation from plants(adapted from [12].with permission of ELSEVIER).

Cellulose is a wide source of raw materials and is the most abundant renewable resource currently. It is a linear polymer material with incomplete crystallization, and is composed of amorphous and crystalline regions. Cellulose materials can be used in primary and secondary packaging. However, their usability is restricted by their lack of ideal barrier properties (against water, oxygen, and ultraviolet) and antibacterial abilities. To solve this problem, the development of high-performance cellulose-based composite materials has become a hot research topic for many scholars. Nanocellulose, a type of nano-material with high crystallinity, possesses superior properties, including renewability, high Young’s modulus, specific surface area, and high tensile strength, and has been reported as an additive in green nanocomposite materials for the food packaging industry [15,16]. Nanocellulose is a kind of cellulose-derived natural fiber at the molecular level, and can be obtained from lignocellulosic materials, marine animals, bacteria, and algae through extraction methods. Differences in extraction techniques and sources of cellulose fibers have significant influence on the characteristics of nanocellulose. Given the abundant sources of plants, nanocellulose extracted from plants seems to be a better and simpler option. Here, a graphical presentation of natural raw material made into nano-scale cellulose is presented in Figure 1 [12].

Normally, nanocellulose can be categorized into three major groups based on its micromorphology, size, and raw material source: cellulose nanocrystals (CNCs), cellulose nanofibrils (CNFs), and bacterial nanocellulose (BNC). CNCs are mainly extracted from cellulosic materials through acid hydrolysis, in which the amorphous region dissociates, and then rod-like nanocrystals with strong acid resistance and high crystallinity will be formed. CNFs, as a type of nano-cellulose, are obtained by a strong mechanical disintegration treatment owing to the complex structure of cellulose fibers [17]. BNC, which is a term for nano-structured cellulose created by bacteria, is cultured in nutrient medium containing carbon sources. Although these categories vary from each other, they are somehow connected in that many nanocellulose sources coexist in an extensive and overlapping material space. The types and characteristics of nanocellulose are unveiled in Table 1.

### 2.2. Preparation of Nanocellulose

Nanocellulose can be prepared in two ways: The first way is the top-down approach, which involves using plant sources, such as wood and byproducts of farm product processing, and handling them with superfine treatment by physical and chemical methods. The second way is the bottom-up approach, which involves using carbon and nitrogen sources of small molecules (such as monosaccharides and hydrolyzed sugars) to synthesize nanocellulose in a culture medium through microbial fermentation or bacterial culture. Overall, differences in the preparation methods of nanocellulose result in differences in crystallinity, morphology, surface chemical properties and mechanical properties, and ultimately, the potential usage of nanocellulose. Table 2 shows the differences among the preparation methods mentioned above.

#### 2.2.1. Chemical Methods

Acid hydrolysis is the most widely used technique for generating CNCs. The equipment used for the acid hydrolysis technique is relatively simple. Moreover, it is the most mature and widely commercialized technique that is currently available. However, in the process of acid hydrolysis, using strong acids, such as sulfuric acid and hydrochloric acid, causes some problems, including strong corrosion of equipment, difficulty in the treatment of waste liquid, and environmental pollution. Although organic acids (oxalic acid, citric acid, and formic acid) and solid acid (phosphotungstic acid) have less influence on equipment and their waste liquid is easier to recycle, their weak acidity results in low reaction efficiency and low product yield, and they require other assistance treatments. Recently, a series of new methods, including 2,2,6,6-tetramethylpiperidinooxy (TEMPO) oxidation, enzymatic hydrolysis, the ionic liquid method, and the deep eutectic solvent method, have been developed to meet the purpose of energy saving and environmental protection. Table 3 lists the novel technologies for extracting CNCs.

#### 2.2.2. Physical Methods

Physical methods are the most common way to prepare CNFs, usually employing external forces such as high-pressure homogenization, high-speed shearing, high-intensity ultrasonication, and high-speed grinding or stirring processing, subjecting plant fibers to shear, cavitation and convective impacts, and destroying the internal structure to obtain nano-scale cellulose. However, mechanical methods demand high energy consumption, requiring approximately 2000–3000 kwh/t [34]. Some scholars have adopted chemical modification and cellulose synthase hydrolysis to break hydrogen bonds and introduced the charge to swell the fibers and reduce the adhesion of amorphous areas, thereby reducing energy consumption. A comparison of the different methods for preparing CNFs is presented in Table 4.

#### 2.2.3. Biological Methods

**Figure 2:**
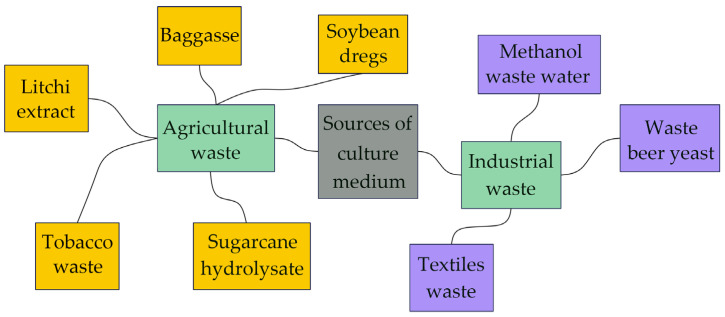
Some carbon sources used as culture medium materials to prepare bacterial cellulose.

The microbial synthesis method is a pattern to generate BNC, and can be mainly divided into static-culture and stirred-culture methods based on fermentation conditions. Different cultivation modes may result in different performances and structures of BNC, as shown in Table 5. The growth of bacteria and the production of bacterial cellulose demand culture medium; therefore, fermentation substrates (culture medium compositions) have a significant effect on the yield and expenditure of bacterial cellulose. Recently, low-cost carbon sources have been developed to optimize the production of bacterial cellulose. Some carbon sources used as culture medium materials for preparing bacterial cellulose are presented in Figure 2.

Ordinarily, static culture is the major mode for generating BNC. Under static conditions, the bacterial biosynthesis of BNC forms a gel film, whereas stirred culture often produces granular or star-like nanocellulose through stirring, shaking, and rotating. No matter what kind of cultivation mode we choose, the synthesis of BNC needs a certain culture medium and appropriate condition (pH, temperature, dissolved oxygen, etc.).

## 3. Preparation of Nanocellulose Composite Films

**Figure 3:**
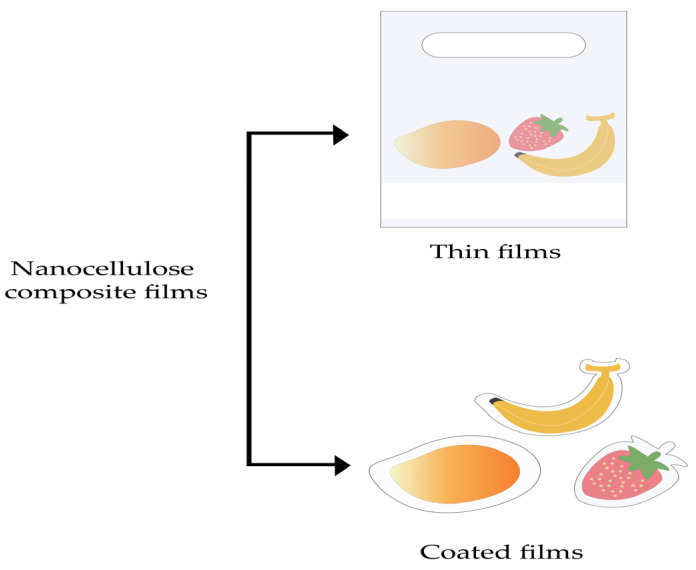
Usage of nanocellulose composite films in food packaging.

**Figure 4:**
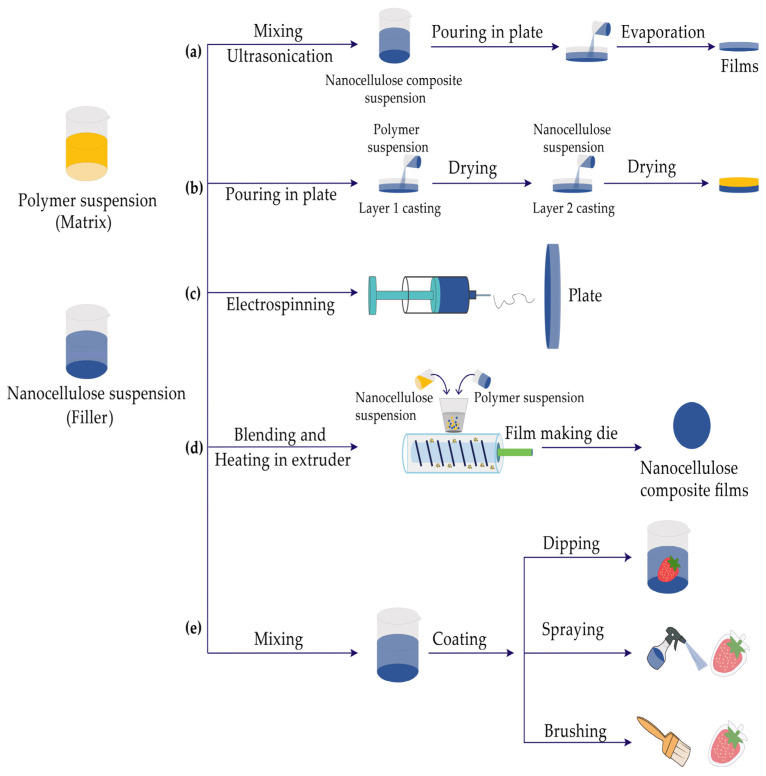
Preparation methods of nanocellulose composite films: (**a**) solvent casting method, (**b**) layer-by-layer assembly, (**c**) electrospinning, (**d**) melt process, (**e**) coating (adapted from [48] with permission of Talyor & Francis).

With the advantages of high specific surface area, high strength, good biocompatibility, natural biodegradability, and easy modification, nanocellulose can be used for drug carriers, cosmetics, optoelectronic materials, food packaging, etc., showing prosperous prospects of application. An increasing number of researchers have shifted from the preparation and characterization of nanocellulose to the development of new materials and enhancement of conventional material properties. On account of its unique morphology and chemical properties, it can be well compounded with other polymer matrices [47], and it is often added as a reinforcing filler to improve the performance of the materials. Nanocellulose composite films used in food packaging can be divided into two major groups: thin films and coated films (Figure 3). The methods used to prepare nanocellulose composite films include wet processes (solution casting, layer-by-layer assembly, and electrospinning), melt processing, and coating, as shown in Figure 4 [48].

### 3.1. Wet Process

Based on the related literature, it can be understood that there are three main ways to form nanocellulose composite films by wet processes: solution casting, layer-by-layer assembly, and electrospinning. These are illustrated in Table 6.

#### 3.1.1. Solution Casting Method

Among these wet processes, the solution casting method is the most common one, and the procedure includes mixing nanocellulose solution and polymer solution, removing the solvent by drying or evaporation, and casting the remainder onto a plate to obtain the product (Figure 4a), which can be divided into two categories according to the solubility of the polymer: aqueous suspension and non-aqueous suspension. In aqueous suspension, nanocellulose will disperse easily to form a stable suspension, and a film will be obtained through mixing, drying, and casting. In non-aqueous suspension, the hydroxyl groups on the surface cannot disperse evenly in solvents. To ensure uniform dispersion in solvents, it is necessary to adopt modification treatments, including alkylation, acetylation, esterification, TEMPO oxidation, polymer grafting, and physical adsorption. By employing the solution casting method, Kang et al. [55] added natural antioxidants to a film-forming matrix consisting of gum arabic and cellulose nanocrystals, and found that composite films can extend the preservation time of raspberries.

#### 3.1.2. Layer-by-Layer Assembly (LBL)

By applying the LBL technique, materials with opposite charges are alternately deposited on the surface of the substrate to form a multilayer film with uniform thickness (Figure 4b). With this method, the properties of the films can also be improved by introducing nanomaterials, biological macromolecules, and conductive polymers, making it one of the most common methods for building composite materials. However, it is not accessible to a wide range of industrial applications, as the process is rather slow and only fits for small samples [16]. Ogonsona et al. [56] utilized hydrogen bonds between cellulose nanocrystal and polyvinyl alcohol to generate composite films by layer-by-layer assembly and found that when the mass fraction of cellulose nanocrystal was 10%, the strength and modulus increased by approximately 415% and 2300%, and oxygen permeation was completely blocked.

#### 3.1.3. Electrospinning

Electrospinning is a method for preparing nanofiber membranes, which involves dissolving polymer materials in organic solvents to prepare a rather dilute solution. The dilute solution is then subjected to a high-voltage electric field to generate strong charges and increase the surface energy. After excessive stretching, polarization and deposition, the processed product is collected on a flat plate and forms closely stacked films with diameters ranging from 2 nm to several micrometers (Figure 4c). With the advantages of high porosity, high specific surface area, more choices of materials, and non-thermal processes, electrospinning is widely used to generate food packaging films [57]. Park et al. [58] prepared poly (vinyl alcohol)/cellulose nanocrystal composites via electrospinning, at a thickness of about 50 μm. Compared with neat poly vinyl alcohol film, the elastic modulus and tensile strength of composite films have increased by 83% and 52.3%, respectively.

### 3.2. Melt Process

Melt processing is a type of processing in which the polymers gradually melt at high temperatures and then will be solidified into the desired shape and thickness through cooling, including extrusion, blow molding, and injection molding. Usually, people use blow molding to prepare beverage bottles and milk bottles, and injection molding to prepare storage boxes and chairs. Undoubtedly, the ordinary way to prepare food packaging films is extrusion, which has high production efficiency and is suitable for mass production, and the schematic diagram of preparing nanocellulose composite films through extrusion is shown in Figure 4d. Owing to the strong hydrophilicity resulting from a large amount of hydroxyl bonds on the surface of nanocellulose, it is hard to disperse in non-polar materials. Meanwhile, the high temperature, high pressure and high shearing within the extruder would destroy the molecular structure and thermal stability. Therefore, it is still challenging to adopt extrusion to develop nanocellulose composite films. To solve this problem, modified treatment should be used to guarantee that the modified nanocellulose can be well dispersed. Moreover, it is necessary to strictly control the temperature to avoid decomposition, which will take place when the temperature reaches approximately 200–300 °C [59].

In recent years, attempts have been made to improve industrial processes through melt processing alongside the increasing attention in this field, focusing on the improvement of dispersion and thermal decomposition temperature [60].

### 3.3. Coating

Nanocellulose composites can be made into coating films [61] and applied on the surface of food to block gases, moisture and grease, thereby prolonging shelf life. In the food industry, coating films play a vital role in preventing water loss, controlling respiration rates, gas exchange, and oxidative browning, maintaining firmness, and prolonging preservation [62]. Composites or polymer solution form a coating film on the surface of food usually via dipping, spraying, and brushing (Figure 4e). Pacaphol et al. [63] prepared an edible coating with CNFs from trunks of hemp, coated spinach leaves with 0.3 and 0.5% *w*/*v* nanocellulose concentrations, and found that the ability of edible film to maintain quality was superior to that of the uncoated spinach.

## 4. Superior Performances of Nanocellulose Composite Films in Food Packaging

Food packaging plays a significant role in the food industry, ensuring food quality in the process of transportation and sales, and prolonging shelf life by preventing microbial contamination, chemical pollution, and blocking water, oxygen, light, external forces, and other unfavorable factors [64,65,66]. Usually, many kinds of materials can be used in food packaging, such as plastic, paper, metal, glass, and ceramics, of which plastic products are most widely employed because of their low cost and stable chemical structure.

Plastic packaging materials amount to over 50 percent of the food packaging market in China. Varieties like polyethylene, polypropylene, polyethylene terephthalate, and polystyren are extensively used [67,68]. Currently, plastic materials are greatly popular due to their strong points, such as their concise production technology, low cost, good plasticity, elasticity, sound insulation, light weight, and excellent corrosion resistance as well as cold and heat resistance, leading to large-scale commercial application. Nevertheless, petroleum-based plastic is generally recognized as a prominent pollutant which is difficult to recycle and degrade. In addition, people are concerned about toxic phthalates, bisphenol A, and other substances contained in plastics, which may migrate to food. Moreover, plastic food packaging accounts for more than 40% of plastic waste for the whole world [69]. Therefore, the development of energy-saving, environmentally friendly and safe food packing materials is one of the top priorities worldwide. With the growing awareness of environmental protection, there is an increasing need for biodegradable petrochemical materials, such as polysaccharides, proteins, lipids, and their synthetic counterparts. Food packaging materials should meet specific requirements, including barrier, thermal and mechanical properties, to ensure the security and quality of food. However, in contrast to synthetic polymers, conventional biopolymers are disadvantaged in terms of these properties [70], failing to be applied in large-scale commercial applications. Nanocellulose, a plentiful and biodegradable natural polymer, has significant advantages in the development of functional food packaging materials and is a competitive candidate for replacing petroleum-based plastic packaging materials. It has been demonstrated that nanocellulose can be used as a reinforcing agent in other film substrates (such as polyvinyl alcohol, whey protein, chitosan, and polylactic acid) to enhance antibacterial ability, barrier properties, and mechanical performances.

### 4.1. Oxygen Barrier Property

**Figure 5:**
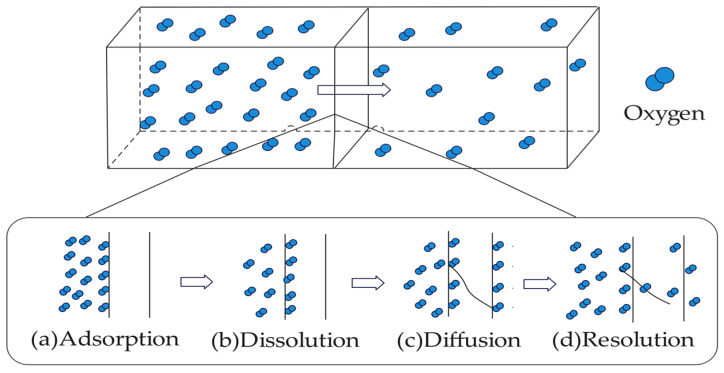
Schematic diagram of oxygen permeation process in films.

**Figure 6:**
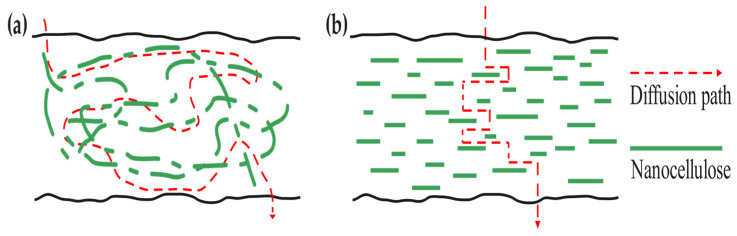
(**a**) Gas diffusion path of CNF; (**b**) gas diffusion path of CNCs through composite films (adapted from [73] with permission of ELSEVIER).

Regarding food preservation, oxygen concentration has a notable impact on food quality, which may induce oxidation, browning, and rancidity. Therefore, maintaining a hypoxic condition helps to extend the shelf life of food. Theoretical research shows that the gas permeation process through membrane materials includes four steps: adsorption, dissolution, diffusion, and resolution [71,72]. The permeation process of oxygen is illustrated in Figure 5. Also, oxygen diffusion varies across different types of nanocellulose due to their unique morphological properties. According to Lu Wang et al. [73], if CNCs are added as a filling material into another polymer matrix, its uniform and rigidly stratified structure will result in fewer gaps in films, increasing the porosity and making the gas diffusion more tortuous, as shown in Figure 6a. When CNF is incorporated with other polymers, its high aspect ratio will form a twist network structure, resulting in a more tortuous diffusion path, as shown in Figure 6b.

The oxygen barrier property of a material is best illustrated by its permeability, including oxygen transmission rate (OTR) and oxygen permeability (OP) [74]. Generally speaking, temperature, pressure, thickness, relative humidity, and morphological properties have significant influence on the oxygen barrier properties. Nanocellulose is a nanoscale material with high crystallization, contains abundant hydroxyl groups, and can form hydrogen bonds with polymer matrices to obtain a close-knit structure which can effectively isolate oxygen.

The reinforcement of nanocellulose with other polymer materials can significantly lower the oxygen transmission rate and oxygen permeability. Mondragon et al. [75] developed bio-nanocomposite films based on a protein matrix, with CNFs and CNCs used as strengthening agents, respectively. The results showed that the addition of CNCs and CNFs into composite films had similar effects. When the additional volumes were 5% and 10%, the oxygen transmittance decreased by 21% and 36%, respectively. Kim et al. [76] prepared a self-standing film containing succinylated cellulose nanofibers and fluoropolymer, and their experiment revealed that oxygen transmittance decreased by about 97% when compared with polyethylene terephthalate membrane. In another study by Kang et al. [77], the oxygen permeability of gum arabic (GA) reinforced with 4% cellulose nanocrystals (CNCs) reduced by 25.30% compared with that of the neat GA films.

### 4.2. Water Vapor Barrier Property

The water vapor barrier property can prevent food from spoilage by absorbing the moisture in the air. Nanocellulose has excellent performance for blocking oxygen, while the water vapor barrier property of its pure film is not ideal for its own hydrophilic characteristics. However, a number of scholars have reported that mixing CNCs with high-polar solvents or matrices, such as starch, guar gum, whey protein, and polyvinyl alcohol, is able to reduce the water vapor transmission rate (WVTR) and water vapor permeability (WVP). This is because the strong hydrogen bonding interaction between the CNCs and the matrix improves the adhesion of the material. Also, the high crystallinity of CNCs has a positive influence on water vapor barrier property since diffusion and adsorption mainly occur in the amorphous region of polymers. Khan et al. [78] prepared nanocrystalline cellulose (NCC) to reinforce chitosan-based biodegradable films, and it was found that the water vapor permeability (WVP) of chitosan/NCC films decreased by 27% when the optimal concentration of NCC was 5% (*w*/*w*). In further experiments, the composite films were exposed to γ irradiation (0.5~50 kGy) and the WVP value decreased by 28.8% at 50 kG. Sukyai et al. [79] extracted CNCs from sugarcane bagasse and incorporated whey protein isolate (WPI) to obtain WPI-based CNC films; they proposed that the addition of CNCs can decrease the water vapor permeability of composite films. The reason why this happens is that CNCs interact with the matrix via a strong hydrogen bond, and with the high crystallinity of CNCs, the water vapor transmission is efficiently cut.

In nonpolar polymer matrices, nanocellulose is difficult to disperse uniformly, so surface hydrophobic modification is often adopted to improve the water vapor barrier performance. Surface adsorption, surface hydroxyl chemical, and graft copolymerization modifications are the most common hydrophobic modification methods for nanocellulose. Aulin et al. [80] incorporated vermiculite nanoplatelets and nanocellulose fibers into biohybrid films through the solvent casting method and found the water vapor barrier property of the biohybrid films was remarkably promoted. Lu P et al. [81] reported that the rate of water vapor transmission in a coated nanofibrillated cellulose (NFC) film amounted to 5088 g/(m^2^ d) (38 °C, 90% RH). However, after adding the hydrophobic substance of beeswax latex into an NFC casting film, the value reduced to 3918 g/(m^2^ d) at the same external condition.

### 4.3. Ultraviolet Barrier Properties

Ultraviolet radiation may lead to the oxidation of lipids, proteins, and vitamins, as well as the degradation of antioxidants, thereby lowering the quality and nutrition of food. Therefore, the ultraviolet barrier abilities of packaging materials are a crucial factor in marketing. There are three possible phenomena when ultraviolet light is exposed to the surface of food packaging film: (a) It may fall on the boundary of films and be reflected directly. (b) It may be absorbed by films and converted into other forms of energy, and then released. (c) It may penetrate the films, react with lipids, accelerate the rancidity of food, destroy vitamins, and induce protein denaturation. Based on the processes mentioned above, two major methods to improve ultraviolet barrier properties are proposed here: (a) Chemical absorbents such as benzophenone and benzotriazole can be employed to absorb ultraviolet radiation. They contain certain toxicants and will degrade under visible light irradiation. (b) Physical blockers can be adopted, including ZnO, TiO_2_, inorganic nanoparticles, and those with low toxicity and higher light stability.

**Figure 7:**
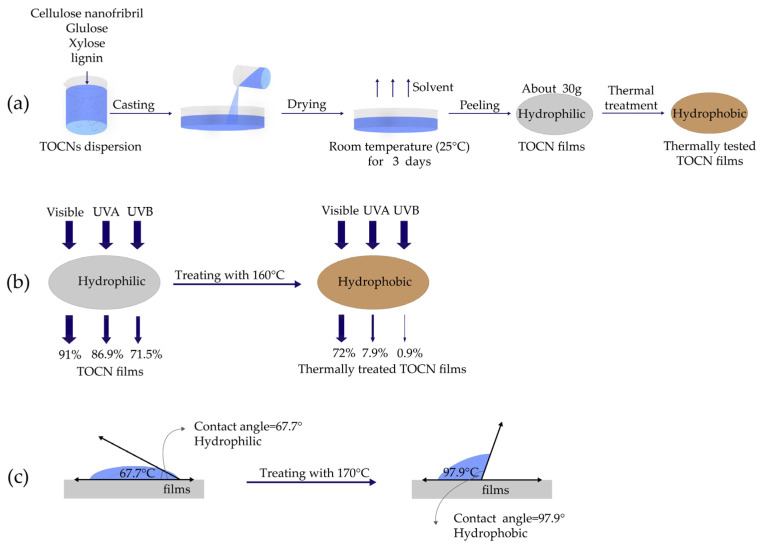
(**a**) Preparation of TOCN UV-blocking films; (**b**) performance changes in thermally treated TOCN films; (**c**) contact angles of water droplets (adapted from [85] with the permission of ELSEVIER).

Feng et al. [82] blended mediated oxidized nanocellulose (ONC), carbon dots (CDs), 2,2,6,6-tetramethyl-1-piperidinyloxy (TEMPO), and zinc oxide (ZnO) nanostructures to obtain highly efficient and transparent UV-blocking nanocellulose films through high-pressured extrusion. In their experiments, with the addition of 4 wt% sheet-like ZnO, the UV-blocking ratio of the composite films at 300 nm and 225 nm was 92.74% and 98.99%, respectively. Some scholars have attempted to improve the ultraviolet (UV) resistance of nanocellulose films through the addition of lignin [83], glycerol [84], heat treatment [85], and other methods. Yang et al. [85] used TEMPO-oxidized cellulose nanofibril (TOCN) to obtain TOCN films via solution casting, as shown in Figure 7a. They applied the wavelength of 555 nm to access the optical properties of the film samples, finding the optical transmittance (T) value of original TOCN films as follows: T (555 nm) was 91.1%, T (ultraviolet A) was 86.9%, and T (ultraviolet B) was 71.5%, indicating high optical transmittance. After a simple thermal treatment at 160 °C, ultraviolet (UV) blocking properties were facilitated directly without using UV absorbent, and the film could fully absorb ultraviolet A and ultraviolet B with visible light transmittance up to 72%, as presented in Figure 7b. They also tested the contact angles of water droplets after thermal treatment at 170 °C, which rose from 67.7° to 97.7°, as presented in Figure 7c, showing the change from hydrophilic to hydrophobic.

### 4.4. Antibacterial Activity

The antibacterial abilities of food packaging films have an essential impact on storage time, so functional films with the abilities to inhibit or kill surface bacteria are crucial for food quality. Nanocellulose naturally has no antibacterial properties, and the antibacterial abilities of composite films are mainly derived from the following two aspects: On the one hand, it can incorporate antibacterial polymers (chitosan, sodium alginate, polyhexamethylene biguanide, polypyrrole, etc.) to obtain new antibacterial composites owing to its high specific surface area and good solubility. On the other hand, its own characteristics are conducive to the addition of antibacterial substances (essential oils, metal nanoparticles, nisin, cinnamaldehyde, glutaraldehyde, pomegranate extract, etc.). Nanocellulose compounded with different types of antibacterial substances results in a difference in antibacterial mechanisms. Common antibacterial mechanisms can be roughly divided into several categories: (a) Positive charge can be assigned to the surface of nanocellulose since antibacterial abilities are positively associated with amount of positive charge. Amination, quaternization, or a compound with positively charged metals, such as chitosan or guar gum, can be employed, which can speed up the reaction between nanocellulose and the negative charge on the bacterial surface, and the integrity of the bacteria will be destroyed to achieve antibacterial purposes. (b) Inorganic antibacterial agents, such as Cu^2+^, Ag^2+^, and Zn^2+^, which can attach to the surface of bacterial and fungal cells, can be used to damage the cell membrane and coagulate protein, thereby reducing the activity of bacteria and fungi. (c) Some natural peptide antibacterial agents, such as lysozyme and bacteriocins, can destroy the bacterial cell membrane and make its glycoprotein decompose when exposed to water, leading to bacteriolysis.

Madivoli et al. [86] used a cellulose nanofibril–polyvinyl alcohol nanocomposite embedded with silver nanoparticles to prepare nanocomposite films using a solvent casting method. In their experiment, the pure polyvinyl alcohol films had no inhibition zones for the bacteria, while the composite films formed strong inhibition zones around the disk, demonstrating the potential for antibacterial packaging materials. Vilela et al. [87] fabricated poly (sulfobetaine methacrylate) (PSBMA) and BNC, added them into freestanding films, and observed that PSBMA/BNC nanocomposite films significantly inhibited the growth and reproduction of pathogenic microorganisms that cause food spoilage and foodborne diseases, demonstrating good prospects in the food packaging field.

Nanocellulose has the features of high specific surface area and good solubility, which are beneficial for the addition of antibacterial substances to inhibit the growth of microorganisms. In recent years, researchers have used natural extractives (tea polyphenols, lauric acid, pomegranate extract) incorporated with nanocellulose to prepare antimicrobial composite films as potential materials for food packaging. Chitosan is one of the most common natural extractives used to improve the antibacterial properties of composite films. Sundaram et al. [88] prepared biodegradable composite films with excellent mechanical strength and antibacterial properties by the addition of CNFs, chitosan, and S-nitroso-N-acetyl-D-penicillamine. The nanocomposite films exhibited strong antibacterial activity against of *Enterococcus faecalis*, *Staphylococcus aureus*, and *Listeria monocytogenes*, suggesting that these nanocomposites are promising choices for food packaging applications. Lauric acid, a natural lipid antimicrobial substance, has positive effects on Gram-positive and Gram-negative bacteria. Zahan et al. [89] adopted BC as raw material and mixed it with lauric acid as an antibacterial agent to obtain a new biodegradable and antimicrobial packaging material. Their research showed that the new material had a significant restraint on Bacillus subtilis, whereas there was no obvious effect on *Escherichia coli*. Hassan et al. [90] successfully prepared antibacterial and antioxidant films and coatings using CNF/pectin/pomegranate extract which showed flexibility, good mechanical properties, and high greaseproof properties. In their study, pomegranate extract acted as a strong antibacterial agent against Gram-positive and Gram-negative bacteria, for it contains several polyphenolic constituents, including chlorogenic acid, catechins, and gallic and ellagic acid.

### 4.5. Mechanical Properties

Mechanical performance is one of the most important indices for evaluating packaging materials, and reflects the application ability of packaging materials. The main measurement parameters reflecting the mechanical performance of packaging films include tensile strength, elongation at break, the modulus, etc. The reinforcement mechanism of nanocellulose on polymers depends mainly on hydrogen bonds, chain entanglement, and the promotion of crystallization. (a) Hydrogen bonds: Nanocellulose can be added as filling material into other polymer matrices, and an extensive hydrogen and ionic bond network will be formed by intermolecular interactions between nanocellulose and the polymer matrix [91]. The plentiful hydrogen bonds form a rigid network system, which increases the value of elasticity and shear strength, thus improving the mechanical properties of the composites. (b) Nanocellulose, especially slender and filamentous CNFs, can entangle with polymer matrix chains to form a three-dimensional network structure with superb stability to promote impact resistance and toughness. (c) Nanocellulose with high crystallinity can be used as a heterogeneous nucleation agent to promote the crystallization process, which is beneficial for improving mechanical properties. Tensile strength has been enhanced by incorporating nanocellulose into polymer matrixes. Khan et al. [78] reinforced nanocrystalline cellulose (NCC) with chitosan-based biodegradable films to develop chitosan/NCC films, and the influence of different contents of NCC on the tensile strength of chitosan/NCC films was also studied, revealing that the optimum content was 5% (*w*/*w*) with an improvement of 26% in tensile strength compared to chitosan films. Yadav et al. [92] reported an improvement in the tensile strength of CNC/chitosan composite films with the addition of 4 wt.% CNCs, indicating a 39% increase. Leppänen et al. [93] prepared hybrid films containing both CNFs and CNCs of 50% that displayed excellent mechanical properties, and found that the addition of CNFs was conducive to the strength of the film.

The incorporation of nanocellulose influences the elongation at break of nanocomposite films. Some authors have observed an increase in elongation at break with the inclusion of nanocellulose. Raj et al. [94] extracted cellulose microfiber from peanut shells, incorporated it in an agar matrix to develop bio-nanocomposite films, and evaluated its potential usage in food packaging applications. They found a 6% improvement in elongation at break in nanocomposite films. Similarly, in the experiment of Kang et al. [77], the incorporation of 4 wt.% CNCs improved the elongation at break from 42.50% to 62.79%. In contrast, a reduction in elongation at break was observed with the increase in nanocellulose content in starch-based composite films by Chen et al. [95], since the flow of the starch molecular chain was restricted by the rigid network formed by nanocellulose.

The modulus of nanocomposites has increased with the addition of nanocellulose. In a study, Nair et al. [96] used CNFs as reinforcing fillers in a bio-based epoxy resin and found that the modulus of toughness of the composites had a remarkable improvement of almost 184 times (0.98 to 2.79 MPa) compared to the neat epoxy. Yadav et al. [92] observed that the Young’s modulus of the chitosan-based composite films increased along with the addition of 4 wt.% CNCs, reaching 78%.

### 4.6. Thermal Stabilities

Thermal stabilities are crucial to the food packaging industry because higher decomposition temperatures can prevent the degradation of production in the process of compounding and extrusion. The thermal degradation of film normally involves two major steps [97]: (a) the liberation of low-molecular-weight compounds such as water; (b) the depolymerization of constituents causing the largest weight loss. It has been demonstrated that the thermal stabilities of nanocomposite films are considerably affected by the matrix, filler, the dispersion of filler, and the molecular bonding between the matrix and the filler. Hence, the addition of nanocellulose as a reinforcing agent in polymeric matrices could remarkably improve thermal stabilities due to its crystalline structure and good interfacial adhesion between the filler and the matrix.

Mandal and Chakrabarty [98] observed an outstanding increase in the thermal properties of NCC/PVOH composites. The strong intermolecular hydrogen bonding between the filler and matrix could enhance the required energy to the dissociate crosslinked PVOH nanocomposites and improve the thermal degradation temperature. According to Ben Cheikh et al. [99], the addition of CNFs could significantly improve the decomposition temperature of composites by incorporating 10% CNFs into a polyvinyl alcohol matrix. Compared with neat polyvinyl alcohol, the thermal decomposition temperature of composites increased from 272.5 °C to 339 °C. Table 7 presents some different nanocomposites which could improve thermal stabilities.

### 4.7. Biodegradation Properties

**Figure 8:**
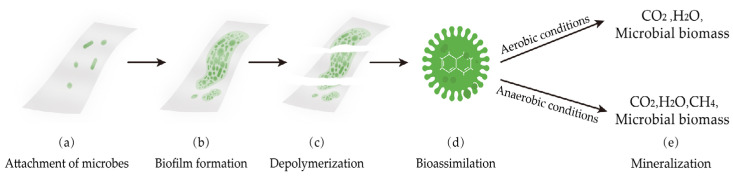
Schematic illustration of polymer biodegradation process (adapted from [107] with the permission of ELSEVIER).

Biodegradability refers to the fact that some materials can be degraded by microorganisms under natural or composting conditions and ultimately mineralize into carbon dioxide (CO_2_) and/or methane (CH_4_), H_2_O, and inorganic salts. Generally, the biodegradation of polymer products can be summarized into five major phases [107], which are schematically shown in Figure 8: (a) the attachment of microbes, gathering of microbes, and adherence to the surface of the polymer; (b) biofilm formation followed by changes in physical and chemical properties along with the increasing attachment of microbes; (c) depolymerization: microbes secrete extracellular enzymes and exopolysaccharides which can fragment the polymer degradation into oligomers, dimers and monomers; (d) bio-assimilation: the decomposed polymer is absorbed and utilized by microbes; (e) mineralization: polymers completely degrade into CO_2_, H_2_O, and microbial biomass under aerobic conditions, while under anaerobic conditions, they degrade into CO_2_, H_2_O, CH_4_, and microbial biomass.

Nanocellulose can not only be made into neat films, but can also be mixed with other biodegradable polymers, such as polylactic acid and polybutylene succinate. It is an ideal food packaging material that can replace petroleum-based products due to its excellent biodegradability.

According to Zahan et al. [89], pure BC film experienced more than 50% degradation by the third day and was completely degraded by the seventh day in the soil. HAI et al. [100] blended bamboo cellulose nanofiber and chitin nanofiber to obtain a green nanocomposite that could fully biodegrade in a week. Arun et al. [108] synthesized a polyvinyl alcohol (PVA)-CNF oil-based composite film through the incorporation of cellulose nanofibers extracted from coconut shells and a polyvinyl alcohol polymer matrix containing oil. They then studied the biodegradability of the developed films for 45 days. On the 45th day, the degradation was up to 87.34 ± 0.91%, exhibiting distinguished biodegradability.

### 4.8. Edible Properties

Natural biomass materials (proteins, polysaccharides, and lipids) can be made into edible films. After being fortified by edible plasticizers, antioxidants, and antibacterial agents, they will experience mixing, heating, coating, drying, and other processes, forming a film with certain physical and chemical properties and a dense structure.

Nanocellulose is a natural polysaccharide that has edible properties. As we can see, there are few neat nanocellulose edible films, owing to their high crystallinity, which makes it hard to gelatinize in water and results in poor film-forming ability. In general, people often used nanocellulose as a reinforcing filler that will be mixed with other matrices to improve the performance of composite edible films. Zhang et al. [109] proposed that most edible packaging films often fail to meet the standards of applications, so people often adopt nanocrystals (CNCs) and cellulose nanofibers (CNFs) as reinforcing fillers in edible films to improve their properties, although there is a key “optimum” concentration for the addition of CNCs and CNFs. Jeevahan et al. [110] extracted nanocellulose from dried bananas and pseudostems, added it into rice-starch-based edible films, and investigated the water vapor permeability, mechanical strength, solubility, and color of composite edible films. The results clearly showed that the additives in starch films significantly improved the properties of edible films, demonstrating that rice-starch-based edible films have promising prospects. Papadaki et al. [111] developed edible films by using whey protein concentrate (WPC) and bacterial cellulose nano-whiskers (BCNW), and the results showed that BCNW can be successfully incorporated and evenly distributed in the WPC film matrix. Meanwhile, in the mechanical properties test, the tensile strength and Young’s modulus of the WPC/BCNW composite films increased by 32% and 80%, respectively. Padrão et al. [112] blended bovine lactoferrin (bLF) with bacterial cellulose films and observed that composite films could reduce the activity of *Escherichia coli* and *Staphylococcus aureus*.

## 5. Applications of Nanocellulose Composite Films in Food Packaging

Food packaging is a crucial part of storage, sales, and transportation in the food industry, protecting food from oxygen, water, dust, and microorganisms, and maintaining the quality, safety, and freshness of food products. Ahankari et al. [70] stated that biopolymers have poor barrier, thermal, and mechanical properties when compared with their synthetic counterparts. The incorporation of nanoparticles to make up for the shortcomings of biopolymer films has been proven to be a promising strategy, among which nanocellulose is an excellent candidate due to its excellent mechanical properties, renewability, and biodegradability [113]. Additionally, it has been revealed that nanocellulose can improve the required properties of food packaging due to its ideal monomer for crosslinking or grafting [70,114].

Recently, the operation of incorporating nanocellulose into biopolymer matrices to obtain nanocellulose composite films with excellent mechanical, barrier, and thermal properties has been widely reported [109,115,116,117]. It can be a remarkable packaging and coating material for extending the shelf life and maintaining the quality of various fruits, vegetables, and meat products. Additionally, the addition of antibacterial substances for the preparation of antimicrobial film composites, which have great potential for food packaging, has become an attractive research topic [118,119,120].

### 5.1. Meat and Meat Products

Meat and meat products are rich in proteins and fats, which are more susceptible to lipid peroxidation and protein oxidation caused by microorganisms, light, and oxygen. Improper packaging methods and storage may result in dip loss, unpleasant flavors, darkening of color, and reduced commercial value. In recent years, nanocellulose composite films have been investigated in the processing and packaging of meat and meat products because of their ability to block water vapor and oxygen, reducing air and moisture contact with products to maintain quality and extend shelf life. Some nanocellulose composite films have been identified by scholars as packaging films for meat and meat products, as shown in Table 8.

In a study by Costa et al. [126], the addition of CNCs improved the thermal stabilities, oxygen barrier properties, tensile strength, and Young’s modulus of chitosan/CNC films. In addition, the films can effectively restrict the reproduction of Gram-positive, Gram-negative, and Candida albicans bacteria and extend shelf life. Compared with commercially available films, composite films can inhibit the propagation of Pseudomonas and Enterobacteriaceae in chicken meat. Meanwhile, total volatile basic nitrogen was reduced, proving the ability to delay meat deterioration under refrigeration conditions. Qian et al. [127] investigated the preservation of chilled meat by blending curdlan with nanocellulose. They found that the mechanical, thermal, and gas barrier properties and transparency of the blended film were superior to those of the curdlan film. After fresh pork was wrapped with the blended films and refrigerated, the lipid oxidation, total volatile basic nitrogen, and microbial growth of meat were measured, showing that the blended film had better antibacterial performance in delaying the spoilage and decomposition of protein, which slowed down the oxidation process and extended the storage time of meat for 12 days.

Nanocellulose, which has high specific surface area and good solubility, is conducive to the addition of antibacterial substances, and will inhibit the growth of microorganisms. In recent decades, the addition of antibacterial substances into composite films to postpone food deterioration has been reported and affirmed by researchers. Salmieri et al. [128] combined poly lactic acid (PLA) with cellulose nanocrystals (CNCs) to develop a new bioactive nanocomposite film through compression molding, added nisin as an antimicrobial agent into the PLA-CNC film, and employed ham as a test sample to measure the antimicrobial resistance of the PLA-CNC-nisin film, declaring a notable decrease in L. monocytogenes from day 1 and a complete inhibition on day 3, which lasted to the 14th day. In a study by Xie et al. [129], a biopolymer-based packaging material was prepared by solution casting, embedding BNC as a reinforcing filler and curcumin as an active antibacterial filler in potato peel powder. The composite film prominently improved the tensile strength and antioxidant properties, and lowered the water vapor permeability, oxygen permeability, and moisture content. The author used fresh pork as the sample and coated it with film for 7 days to test its practical application, proving that the lipid oxidation was reduced with less malondialdehyde (MDA).

### 5.2. Fruits and Vegetables

Physiological and biochemical events, including transpiration and respiration, still occur in fruits and vegetables after harvest. In addition, the invasion of foreign microorganisms and oxidative damage will accelerate the speed of self-decomposition and water loss, reduce sensory quality and nutritional value, and shorten shelf life. Sound food packaging is crucial for maintaining the commercial value (e.g., appearance and color) of fruits and vegetables by inhibiting weight loss and respiratory rate, reducing exposure to oxygen, and preventing fungal and bacterial infection [130]. Some nanocellulose composite films have been identified by scholars as packaging films for fruits and vegetables, as listed in Table 9.

Li et al. [138] aligned cellulose nanocrystals (CNCs) and cellulose nanofibrils (CNFs) in polyvinyl alcohol (PVA) to obtain CNC/PVA and CNF/PVA films, and then used an external magnetic field (MF) to ensure effective dispersion. The results showed that the mechanical, barrier, and hydrophobic properties, as well as thermal stability, increased. The oxygen barrier property of PVA/CNC-MF was superior to that of PVA/CNF-MF in MF-treated nanocomposite films. Meanwhile, the author adopted strawberries with a high respiration rate as samples to evaluate its usability and analyzed the color difference (ΔE) after storage for six days, detecting that the ΔE values of composite films were notably lower than those of the control group (unpacked and packed with PE film). They proposed that PVA/nanocellulose films have good oxygen barrier properties for achieving color retention.

Kang et al. [77] prepared a new bio-nanocomposite film using cellulose nanocrystals (CNCs) as the reinforcing material and gum arabic (GA) as the matrix, finding that when the content of nanocellulose was 4% it could effectively improve the tensile strength and elongation at break, which increased by 2.21 MPa and 62.79%, respectively. In addition, the water vapor permeability and oxygen barrier capacity decreased by 10.61% and 25.30%, respectively. They assessed the preservation effect of the composite film on fresh strawberries at 50% relative humidity and 4 °C over 9 days, unveiling that the weight loss of strawberries decreased by 23.80% and the value of firmness, total color difference, and total soluble solids were more positive than those of the control group, which verified that the film can slow down the spoilage of strawberries during storage.

Ponni et al. [139] extracted nano-fibrillated cellulose from banana pseudostem, compounded it with polyvinyl alcohol and polyacrylic acid to develop a novel nano-film by a solvent casting method, and measured its physical and biochemical parameters. They found that the nano-film had many excellent properties, including UV protection, strong cross-linking, oxygen barrier capacity, and thermal stability. Taking the gas barrier capacities as an example, the oxygen and carbon dioxide transmittances of the conventional film were 21,800 mL/ m^2^/day and 26,500 mL/ m^2^/day, respectively, while those of nano-film were 498 mL/m^2^/day and 821 mL/m^2^/day, indicating that the nano-film had excellent gas barrier properties. Also, they selected tomato fruits to verify the protective quality, demonstrating that the firmness, total soluble solids, tillable acidity, and ascorbic acid (mg/100 g) were superior to those of the conventional film and control (unwrapped) group, and the physiological weight loss (%), pH, total sugar (%), and reduced sugar (%) were lower than another two groups. Moreover, the shelf life of potato with nano-film was longer than that with the conventional film, extending it by 7 days.

Garg et al. [140] developed an eco-friendly biodegradable film by mixing guar gum, surface-modified crystalline nanocellulose, polyethylene glycol, and a cross-linker glutaraldehyde, and tested its antibacterial activity, finding that the composite film could significantly restrain the growth of Gram-positive (*S. aureus* and *B. subtilis*) and Gram-negative (*E. coli*) bacteria. Then, they used fresh grapes as samples to assess packaging efficiency. They were packed in composite film and stored in open air for 30 days. On the seventh day, the physical appearance of the grapes coated with the film showed little difference, while that of the control group experienced shrinking in size. Microbial growth was obvious in the control group, whereas only small shrinkage was observed in the packed grapes after 15 days. Until the 30th day, the author did not observe any growth of microorganisms on the packed grapes.

### 5.3. Aquatic Products

Aquatic products, which are rich in proteins, amino acids, vitamins, and fats, act as an indispensable source of nutrition for human beings. However, they contain not only a large amount of water in vivo and in vitro, but also active endogenous proteins. As a result, unsaturated fatty acids may be oxidized to produce aldehydes and ketones, giving rise to undesirable flavors [141]. By selecting appropriate packaging materials, the rates of oxidation, enzymatic action, and microbiological action can be slowed down.

Recently, it has been reported that intelligent food packaging has been developed to monitor the freshness of aquatic products. Zhou et al. [142] designed chitosan/bacterial cellulose-based films, incorporating tea polyphenol nanoliposomes (antibacterial agent) via the casting method. Fish fillets were wrapped with the film and the total volatile basic nitrogen (TVB-N) value was measured to detect microbial growth and enzymatic activity. The experiment showed that the fish fillets wrapped with film had higher TVB-N, but this was markedly inhibited after 7 days of storage. Compared with the control group, TVB-N was reduced by 49.56% on the 12th day. This phenomenon may be ascribed to the continuous release of tea polyphenol nanoliposomes. Wen et al. [143] developed an intelligent/active food packaging film incorporated with TEMPO-oxidized bacterial cellulose (TOBC), thymol (THY), and anthocyanin-rich purple potato extract (ANT) to extend the storage time of shrimp. They reported that ANT is allergic to pH and has a colorimetric response to volatile ammonia, which can be detected by the naked eye, making it accessible to monitor the freshness of shrimp. THY is treated as a natural antibacterial agent to improve the antibacterial and antioxidant activities of composite films. After three experiments were conducted, the film still maintained good antioxidant, antibacterial, and color response performances, demonstrating its bright future in the real-time monitoring of freshness.

In another study, Li et al. [144] investigated three types of nanocellulose prepared by ulfuric acid, citric acid, and TEMPO, respectively, for monitoring the freshness of shrimp. Nanocellulose incorporated with PVA and anthocyanin (AH) serves as matrix and indicator, respectively. The research showed that these three types of nanocellulose have remarkably improved mechanical and hydrophobic properties, as well as thermal stability, enhancing the sensitivity of the PVA/AH smart films to pH. By applying the films to monitor the freshness of shrimp, the extent of freshness (fresh, sub-fresh, and spoiled) can be easily distinguished via the change in color.

### 5.4. Edible Films in Food Products

Edible film is an environmentally friendly packaging material, meeting the requirements of food packaging with edible properties, biocompatibility, barrier capacity, and non-toxic and pollution-free features. Regarding the development of edible films for food packaging, nanocellulose has gained tremendous attention due to its various beneficial attributes in recent years.

Pacaphol et al. [63] investigated the quality of spinach coated with 0.3 and 0.5% *w*/*v* CNF suspensions over 3 days of storage at 25 °C. Compared with uncoated spinach leaves, the coating delayed the color change and maintained the chlorophyll, appearance, and moisture content. In addition, the coated spinach samples exhibited a considerable decrease in respiration rate. These findings indicated that edible nanocellulose films can be applied to green vegetables to extend their shelf life after harvest. Ghosh et al. [145] designed an edible film with CNFs, chitosan, and curcumin using a single-step co-precipitation method and investigated the effect of coating on kiwifruits by measuring firmness, weight, respiration rate, total soluble solids, and other properties for 10 days at 10 °C preservation. The practical application displayed that edible coating materials could be successfully used as packaging materials during storage. Padrão et al. [112] blended bovine lactoferrin with bacterial bellulose films and observed that the composite films could reduce the activity of *Escherichia coli* and *Staphylococcus aureus*.

## 6. Security Assessment of Nanocellulose Composite Films

As a type of food packaging material, the security issues associated with nanocellulose composite films cannot be ignored, especially when they are employed to produce edible films, fruit coatings, or react with chemical reagents to prepare high-performance composite films. The safety assessment of food packaging materials mainly includes two aspects: migration and biotoxicity assessments. Packaging materials are in direct contact with food, so the composition of the raw material may migrate into the food. Undoubtedly, natural cellulose is innocuous, and its derivatives are considered to be safe. Moreover, micron-sized cellulose and its derivatives have already been adopted as fillers and thickeners in food processing, and are generally regarded as safe for ingestion [146]. However, nanocellulose is yet to be considered as safe or accepted as a food ingredient, since the extremely small size of nanocellulose means that it may invade human organs and cells, interact with biological systems, and remain in the human body, threatening human health [147,148]. Therefore, it is necessary to conduct further research to evaluate the safety of nanocellulose composite films, especially those adopted as edible films and fruit coatings. A biotoxicity assessment assesses the cytotoxicity and genotoxicity of a packaging material to evaluate the potential health risks to the human body through in vivo and in vitro experiments. Before the commercial use of nanocellulose for food packaging, further research on cytotoxicity is imperative to assure that there is no growth inhibition, cytolysis, or death in the cells of the body. Also, experiments on DNA damage and mutation induction are required to ensure that there are no potential genetic effects of nanocellulose materials. The in vitro cytotoxic and genotoxic properties of nanocellulose made from birch pulp were once tested by Pitkanen et al. [149] and the results displayed nothing about DNA damage. Recently, a limited number of investigations into the toxicity of nanocellulose in both in vitro and in vivo models have suggested that nanocellulose does not create cytotoxic or genetic injuries [150,151]. However, there is still a data gap. The unrepresentative exposure methods, high dose, short-duration testing, the absence of a response analysis, and a lack of control groups all make it difficult to draw a conclusion that nanocellulose is safe. Additionally, few toxicological properties of nanocellulose composite films have been reported considering its influence on human health.

The evaluation of ecotoxicity is also an important component of safety assessments to assesses the behavior and effects of nanocellulose composite films in the ecosystem, including the impacts on plants, microorganisms, animals, and the environment. Food packaging materials possessing excellent biodegradability and non-ecotoxic properties would be ideal candidates. Arvidsson et al. [152] adopted seven advanced materials (graphene, graphene oxide, nanocellulose, nano-sized molybdenum disulfide, and MXenes) and screened their environmental threats based on aquatic ecotoxicity and production volume. It was unveiled that only MXenes showed high aquatic ecotoxicity. On the whole, studies on the ecotoxicity of nanocellulose composites are rare.

In general, there are scarce reports on the migration, biotoxicity, and ecotoxicity of nanocellulose, and most of these issues are not specific to nanocellulose composite films. Countries such as the United States, Japan, Canada, and Finland have incorporated nanocellulose into their national new material development strategies, but we have not found any regulations or standards for usage or the commercialization of nanocellulose-based composites for food packaging. This phenomenon is largely caused by the absence of migration, biotoxicity, and ecotoxicity assessments on nanocellulose composite films and the gap in understanding on their potential health risks to humans. Owing to the current data gaps and limited safety assessments of nanocellulose composite films, internationally standardized methods and more high-quality studies are urgently required to help us to obtain more unambiguous information about the safety impacts of nanocellulose, as well establish relevant standards and legislation for nanocellulose composite films across the world.

## 7. Conclusion and Future Prospects

Traditional petroleum-based packaging materials can no longer meet the needs of sustainable development. Nanocellulose could replace petroleum-based materials due to its promising advantages, including rich sources, biodegradability, excellent mechanical properties, biocompatibility, etc. Different types of nanocellulose (CNCs, CNFs, and BNC) can be extracted from different sources to obtain desirable characteristics for the preservation of specific products. The techniques to formulate nanocellulose composite films include solution casting, layer-by-layer assembly, electrospinning, melt processing, and coating, which bring about different properties. Nanocellulose is often used as a reinforcing agent in other film substrates to enhance barrier properties, UV shielding abilities, thermal stabilities, and mechanical, biodegradable, and edible properties in the food industry, demonstrating its great potential for application in the food packaging industry. Nanocellulose composite films and coatings can extend shelf life and maintain the quality of diverse food products such as fruits, vegetables, and meats, as has already been reported by some relevant studies. However, it is still hard to transfer these from the laboratory to the market, and the outlook on the preparation and application of nanocellulose composite films has been described in the following ways: (a) The preparation method should be optimized to reduce the cost when nanocellulose is incorporated with other polymers to provide theoretical guidance for large-scale production. (b) Efforts should be made to find a key addition ratio when combining nanocellulose with other materials to obtain high-performance composite films. (c) Edible nanocellulose composite films as the inner packaging of preserved fruit, pastries, instant soups, and other convenience foods could be developed. (d) A comprehensive evaluation of the safety (migration, biotoxicity, ecotoxicity) of nanocellulose composite films should be conducted before large-scale commercial application.

## Figures and Tables

**Table 1:** Types and characteristics of nanocellulose.

Types	Other Nomenclature	Micromorphology	Typical Sources	Average Size	Preparation Method	Ref.
Cellulose nanocrystals (CNCs)	Cellulose whiskers (CNWs), cellulose nanocrystalline (CNC), nanocrystalline cellulose (NCC)	Whiskers, rod-shaped	Plants (wood, cotton, hemp, flax, etc.)	Diameter: 5–70 nm Length: 100–250 nm	Acid hydrolysis	[18,19]
Cellulose nanofibrils (CNFs)	Micro-fibrillated cellulose (MFC), nano-fibrillated cellulose (NFC)	Twisted filamentous	Plants (wood, beet, cotton, hemp, flax, etc.)	Diameter: 5–60 nm Length: several micrometers	Mechanical treatment	[20,21]
Bacterial nanocellulose (BNC)	Bacterial cellulose (BC), regenerated bacterial celllose(RBC)	Gel-like, ribbon-like	Acetobacter, xylinum, pasteurii, etc.	Diameter: 20–100 nm Length: unfixed	Bacterial synthesis	[7,22]

**Table 2:** Classification of preparation methods of nanocellulose.

Preparation Methods	Main Sources	Treatment	Advantages	Shortcomings	Product	Ref.
Top-down approach	Plant sources, by-products of agricultural products	Physical methods	Easy operation, mature technology	Environmentally unfriendly	Cellulose nanocrystals (CNCs)	[23,24]
		Chemical methods	Easy operation, easy control	Uneven product size, damage the structure	Cellulose nanofibrils (CNFs)	[23,24]
Bottom-up approach	Culture medium containing carbon sources	Biological methods	Without other ingredients, high purity	Low yield, time consuming	Bacterial nanocellulose (BNC)	[25,26]

**Table 3:** Some new technologies for extracting CNCs and their advantages and disadvantages.

Method	Mechanism	Advantages	Shortcomings	Ref.
TEMPO method	The hydroxyl groups of the cellulose are oxidized to carboxylic groups by strong oxidizing agents, weakening the binding force between fiber molecules and making them more prone to micro-fibrillation	Mild conditions, low environmental contamination, good operability	Costly (reagents are expensive), incomplete reaction	[27,28]
Ionic liquid method	Anions in ionic liquids and hydroxyl groups of cellulose will interact, dissolving cellulose effectively and separating the cellulose chain	Good thermal stability and solubility, recyclable	High cost for recycling	[29,30]
Deep eutectic solvent method	The eutectic mixture can destroy the hydrogen bonds inside cellulose so as to degrade it	Biodegradability, recyclable, low cost, low melting point	Weak swelling ability and solubility of product	[30,31]
Enzymatic hydrolysis	Enzymatic reaction takes place between cellulose hydrolases and the amorphous region of cellulose, increasing the proportion of the crystallization zone	Pollution-free, low energy consumption	Low efficiency	[32,33]

**Table 4:** Comparison of different methods for preparing CNFs.

Method	Conditions	Advantages	Shortcomings	Ref.
High-pressure homogenization	The energy produced by high-pressure and high-speed motion is used to break up the tight structure of the fiber, thereby gradually reducing the fiber size to the nanoscale	Good dispersibility	High energy consumption, the machine is easy to clog up	[35,36]
Microgrinding	Shear forces produced by grinding machine and grindstone are employed to split up hydrogen bonds to decrease the size of cellulosic material	Mass production, Low cost	Inefficiency, changes the crystallinity	[37,38]
High-intensity ultrasonication	The shock wave generated by ultrasound can be exploited to destroy fibrous tissue	Good thermal stability	Low production yield	[39,40]
High-pressure micro-fluidization	High pressure can be deployed to inject fiber suspension through micro channels, and then the fibers will be discharged through the channel, with the shear forces among them promoting nano-fibrillation	High efficiency	Decrease the crystallinity	[41,42]
Steam explosion	The tremendous explosive force produced by the release of high-pressure steam in a short time helps destroy the fiber structure and produce nanofibers of cellulose	Less use of chemical reagents, high efficiency	Need specific equipment	[43,44]
Cryocrushing	Extreme temperature reduction is conducted to embrittle the interior of the fiber, and then fiber fibrillation is realized	Improve heat stability	High cost for equipment	[24,39]

**Table 5:** Methods for preparing BNC.

Method	Significant	Product Morphology	Properties	Advantages	Shortcomings	Ref.
Static culture	Incubator and culture medium are both in a static state	Gel-like	Higher in crystallinity, polymerization, fracture strength, Young’s modulus	Thickness and shape could be controlled	Time-consuming, space-occupying	[45,46]
Stirred culture	Continuous oxygen is input into the culture medium	Granular, star-like	Lower in crystallization, polymerization, fracture strength, Young’s modulus	Good water absorption, rehydration properties	Low yield	[45,46]

**Table 6:** Preparation methods of nanocellulose composite films through wet processes.

Process	Mechanism	Advantages	Shortcomings	Ref.
Solvent casting method	Nanocellulose and polymer are mixed in a corresponding solvent, and then the mixed suspension is cast on a flat plate. After the solvent evaporates, a uniform film takes shape	Nano-film is well distributed and the thickness is easy to control	Time-consuming, solvent needs to be recycled	[49,50]
Layer-by-layer assembly (LBL)	Polymers with opposite charges continuously deposit on the substrate through electrostatic interaction to form a multilayer film with uniform thickness	The composition, thickness, and structure of the film can be controllable at the molecular level	Complicated procedures, large-scale equipment is required	[51,52]
Electrospinning	By using a high-voltage electrostatic field, the polymer solution is stretched and overcomes the surface tension to form a jet stream, and then the solvent will evaporate. Finally, a nanoscale thin film is collected on the receiving device	Process is controllable, bioactive substances can be embedded easily	Complicated process, low yield	[53,54]

**Table 7:** Thermal stabilities of different nanocellulose composites.

Type of NC	NC source	Type of Matrix	Preparation Method	Finding	Ref.
Cellulose nanofiber (CNF)	Bamboo	Chitin nanofiber	Blending	It has better thermal stability than pure CNF	[100]
Cellulose nanocrystals (CNCs)	Water hyacinth stem fiber	Polyvinyl alcohol, gelatin	Casting method	Degradation temperature rises from 380 °C to 385 °C	[101]
Cellulose nanofibrils (CNFs)	Waste coconut husk	Poly(vinyl alcohol) (PVA)	Solution casting	CNF-reinforced films have higher stability than pure PVA film	[102]
Cellulose nanocrystals (CNCs)	Pea hull	Carboxymethyl cellulose(CMC)	Solution casting	The addition of CNCs into CMC increases the melting temperature	[103]
Cellulose nanocrystals (CNCs)	Rice straw	Poly(vinyl alcohol) (PVA), Chitosan(CS)	Solution casting and evaporation technique	CNCs have a positive effect on the improvement in thermal stability	[104]
Bacterial cellulose (BNCs)	Komagataeibacter hansenii	Konjac glucomannan	Solution casting	The addition of BNC increases thermal stability	[105]
Cellulose nanofibers (CNF) and nanocrystals (CNCs)	Commercial product	Gelatine	Casting method	The degradation temperature increases by 7–9 °C	[75]
Cellulose nanofibers (CNFs)	Laboratory	Polyproppylene	Layer-by-layer assembly	The composite films need more energy to melt	[106]

**Table 8:** Nanocellulose composite films for food packaging applications.

Compositions	Application	Findings	Ref.
Bacterial cellulose/chitosan/gelatin/probiotic bacteria	Chicken fillets	Slower rate of oxidative and microbial spoilage	[121]
Bacterial cellulose/polypyrrole/zinc oxide (ZnO)	Chicken thigh meat	Containment of the growth of microbial load Control the increase in pH Increase the shelf life	[122]
Cortex phellodendri/soybean protein/nano-cellulose crystals	Beef tallow	Long-term protection Lowest peroxide value Delay of lipid oxidation	[123]
Polylactic acid (PLA)/ziziphora clinopodioide essential oil/cellulose nanoparticle	Minced beef	Lower total volatile base nitrogen and peroxide value than control samples Decrease in microbial population	[124]
Cellulose nanofiber/whey protein/TiO_2_ /rosemary essential oil	Lamb meat	Extension of the shelf life from about 6 to 15 days Reduction in microbial growth, lipid oxidation, and lipolysis	[125]

**Table 9:** Nanocellulose composite films for the packaging of fruits and vegetables.

Nanocomposite Formulation	Application	Findings	Ref.
Cellulose nanofibers/cinnamon essential oil	Strawberry	Lower weight loss Stronger preservation ability No fungal invasion	[131]
Starch-nanocellulose/polyhexamethylene biguanide	Grape	Delay of the spoilage Extension of shelf life	[132]
Cellulose nanocrystals/chitosan nanoparticle/poly vinyl alcohol	Mango	Prevention of invasion from mango-associated post-harvest pathogens	[133]
konjac glucomannan/zein nanoparticles/ nanocellulose/nano-TiO_2_/nano-SiO_2_	Cherry tomato	Lower reduction in weight loss and firmness Less quality changes Longer shelf life	[134]
Bacterial cellulose/nano-TiO_2_/copper oxide (CuO)	Fresh-cut pepper	Inhibition of the softening, reddening, browning, and rotting of products Excellent anti-fogging property	[135]
Cellulose nanocrystals/gellan gum/gallic acid	Mushroom	Lower color change Antioxidant properties to prevent ripening	[136]
Chitosan/cellulose nanofiber/γ-cyclodextrin/curcumin	Banana, tomato and cut apple slices	Reduction in microbial invasion and water loss Decrease in the rate of oxidation Extension of shelf life	[137]

## Data Availability

The data that support the findings of this study are available from the corresponding author upon reasonable request.
